# Rapid Cure of Buboes

**Published:** 1871-04

**Authors:** 


Rapid Cure of Buboes—Dr. J. Grunfeld, assistant to Sigmond, of Vienna, has had much success in extracting the pus by means of a hypodermic needle, India-rubber tube and syringe. Where the cavity fills again, a second operation of the same kind should be undertaken; and when the pus is unhealthy, weak solutions, either of carbolic acid or chlorate of potash, should be injected, and pumped out again by the same syringe. Such patients as were so treated left the hospital much sooner than those whose buboes had been freely laid open.—*Ibid.*

